# Leptospirosis: The “mysterious” mimic

**DOI:** 10.4103/0974-2700.40573

**Published:** 2008

**Authors:** Ricardo Izurieta, Sagar Galwankar, Angela Clem

**Affiliations:** 1 Dr. Donald L. Price Center for Parasite Repository and Education, Tampa, FL, USA; 2 Department of Global Health, University of South Florida, 13201 Bruce B. Downs Blvd., MDC 56, Tampa, FL 33612, USA

**Keywords:** India, *Leptospira*, leptospirosis

## Abstract

Leptospirosis is a potentially fatal bacterial disease that can display a wide array of clinical presentations thus mimicking better-known illnesses. Although, leptospirosis is primarily a zoonotic disease, it frequently inflicts severe illness and death on communities around the globe. A comprehensive overview of the disease in wake of the 2006 outbreaks in India is hereby presented and discussed.

## INTRODUCTION

Climate can have a significant effect on the occurrence of disease. For example, the cyclic pattern of spring and summer rains can increase the incidence of water-borne diseases; and, this annual pattern of rains can be further exacerbated by natural disasters. For example, there was an outbreak of leptospirosis in 2006 that occurred in Mumbai due to significant flooding from torrential rains. Due to its nonspecific symptoms that mimic better-known diseases, leptospirosis has been frequently underdiagnosed and underreported. However, with better reporting and improved diagnostic techniques, “increasing” cases of leptospirosis arebeing detected; and, leptospirosis is now considered an emerging disease.[1]

## 2006 OUTBREAKS IN INDIA

As early as January 2006, there were already 258 individuals diagnosed with leptospirosis in the Siddammanahalli village in the Bellary district. These cases were attributed to a lack of proper drainage and the ingestion of contaminated water.[2] From May 2006 to June 2006, the onslaught of leptospirosis cases continued. There were 40 suspected cases, 10 confirmed cases, and at least one death due to leptospirosis in southern Kerala.[3]

On July 21, 2006, the media reported 14 deaths in Mumbai within a 24-h period due to leptospirosis. These deaths from leptospirosis were just a portion of over 40 deaths from monsoon-related diseases that had occurred in the previous 15 days.[4] To prevent a wider outbreak of leptospirosis in Mumbai similar to a leptospirosis outbreak in 2005 in Sangli district, the authorities curtailed the activities of street vendors selling food in Mumbai to stop the sale of potentially contaminated food and water.[5] By the end of July 2006, over 150 people were suspected to have leptospirosis in Mumbai.[6]

Then, in August of 2006, there were at least eight deaths and more than 17 cases of the pulmonary form of leptospirosis in Surat. The pulmonary form of leptospirosis is characterized as a hemorrhagic pneumonia that can resemble pneumonic plague and hantavirus pulmonary syndrome.[7]

## CAUSATIVE AGENT OF LEPTOSPIROSIS

Leptospirosis is caused by leptospires, which are spiral-shaped bacteria from the family *Leptospiraceae* and genus *Leptospira*. These bacteria are long, thin, and motile spirochetes that can be either free living in the environment or found as parasites in animal hosts.[8]

Leptospires require moist environments for survival. They can survive in contaminated freshwater sources (lakes and ponds) and muddy environments for many months. However, they can only survive for a few hours in saltwater.[9]

The presence of end hooks differentiates leptospires from other spirochetes. There are both saprophytic and pathogenic leptospires present in nature; however, the saprophytic leptospires are not known to cause disease but can frequently contaminate clinical samples of pathogenic leptospires.[1,10]

## CLASSIFICATION OF LEPTOSPIRES

Because of the diversity of leptospires, there are two major methods of classifying them. Leptospires may be divided into serovars (and serogroups) and/or into species.

Leptospires are classified into serovars upon the basis of antigenic characteristics determined by the cross agglutination absorption test. Serovars with similar antigenic characteristics are classified into serogroups.

Leptospires are very antigenically complex and there are over 200 known pathogenic serovars that have been classified into 25 serogroups.[1] Certain serovars have preferences for particular animal host species and usually replicate relatively asymptomatically in the renal tubles of these preferred animal hosts (especially in the adult animals).[1] These animals then become reservoirs for the leptospires; however, leptospires can readily adapt to new host species.[1,11]

Leptospires have also been classified into two species, the pathogenic *Leptospira interrogans* and the saprophytic *Leptospira biflexa*. Recently, more species of leptospires have been recognized; and, strains of leptospires belonging to the same serovar may actually be classified into different species.[1] Frequently, particular geographic areas will have certain strains of leptospires; however, a geographic area cannot be used as an absolute indicator for identification of a particular strain of leptospire.[8]

## ANIMAL RESERVOIRS OF LEPTOSPIRES

Although, leptospirosis is typically a zoonotic disease, leptospires can also infect humans. These bacteria can infect and colonize a wide variety of wild and domestic animals. However, the rat is typically the reservoir of leptospires [Table 1].[1,9,12–15]

**Table 1:** Potential animal reservoirs of leptospires

Horses	Primates
Pigs	Raccoons
Cattle	Stunks
Domestic and wild dogs	Squirrels
Domestic and feral cats	Insectivores (moles, shrews, hedgehogs)
Sheep	Opossums
Goats	Deer
Buffalo	Mice
Mink	Nutria
Reptiles	Hamsters
Amphibians	Silver foxes
Fish	Sea lions
Young birds	Marsupials
Rats	-

Certain serovars of leptospires are typically associated with particular animal species; however, other leptospiral serovars which do not normally reside in a particular animal can produce very severe disease in that animal.[1]

For example, the serovars canicola, icterohemorrhagiae, grippotyphosa, and pomona commonly replicate in dogs; although, dogs also may be occasional hosts for the serovar bratislava.

The serovars hardjobovis, pomona, grippotyphosa, and icterohemorrhagiae frequently replicate in cattle and deer; however, the serovars australis, autumnalis, canicola, bataviae, hebdomadis, krematosis, tarassovi, sejroe, and bratislava may also colonize cattle and deer.[14]

Other sources have compared the severity of serovars hardjo and pomona in cattle. Serovar hardjo infection is usually less severe than serovar pomona in cattle because serovar pomona is more adapted to pigs. Because of the potential severity of pomona, it is usually recommended that unvaccinated cattle remain isolated from pigs.[16]

However, infections with serovar hardjo can and frequently do cause abortion in cattle during the second half of the pregnancy (from 5 months until full-term). In addition, it has been found that cattle are more likely to become infected with leptospirosis, especially serovar hardjo, if there are sheep present on the farm. Therefore, it is recommended that cattle and sheep not graze in the same pastures.[17]

Pigs are frequently host animals for the serovars pomona, bratislava, canicola, tarassovi, and icterohemorrhagiae; however, the serovars grippotyphosa and sejroe may also reside in pigs.[14] Pigs can excrete very large amounts of leptospires in the carrier state (i.e., chronic leptospiral colonization of the renal tubles) and can be important source of human infection. Adult pigs rarely exhibit disease with a leptospiral infection; and, amazingly, the only sign of infection may be fetal death in gestating pigs. In contrast, infant pigs may become severely ill and die.[16]

Rats and mice are commonly colonized by the serovars ballum and icterohemorrhagiae. These serovars can be problematic for humans dealing with rodent infestations.[14]

Sheep can be hosts for the serovars pomona, grippotyphosa, bratislava, and hardjo. Sheep rarely show infection and any illness is predominantly in young or pregnant animals. Approximately 15-20% of abortions in sheep and goats are due to leptospiral infection. Although there is a vaccine available for sheep, it is seldom used unless there is an outbreak of abortions in the flock.[16]

Finally, horses can be the host animals for the serovars pomona, bratislava, canicola, icterohemorrhagiae, and sejroe.[14] Horses are rarely infected by leptospires and the disease is usually mild. Infrequently, infection may result in fetal abortion and deaths of newborn foals. Several months after acute infection, horses may develop periodic opthalmia (iridocyclitis or ‘moon blindness’) which can occur in approximately 50% of cases.[16] There are currently no vaccines for horses; although, cattle vaccines have been used off-label to provide some protect to horses.[16]

In contrast, leptospires rarely colonize certain animals. For example, both domestic and feral cats are infrequently infected and seldom remain infectious with leptospires in the natural environment; although, infections can be induced experimentally. This “immunity” is likely due to the cat's frequent exposure to (infected) rats and mice.[14] Further, cats rarely excrete leptospires in their urine for a prolonged period which means there is a reduced likelihood of transmission to other animals and humans. It has been estimated that 5-10% of domesticated cats in the United Kingdom have antibodies to one or more leptospires. This is comparable to the estimated carrier state of rats in the United Kingdom of 10-15%.[16]

## EPIDEMIOLOGY OF LEPTOSPIROSIS

Leptospirosis occurs throughout the world and is considered the most widespread zoonosis in the world. Further, leptospirosis is endemic in many tropical countries.[8] Most cases occur in the warm, rainy season in the tropics and during the late summer, and early fall in Western countries.[9]

Cases of leptospirosis occurring in tropical and subtropical areas are primarily due to agricultural practices and inadequate housing and sanitation.[18] Leptospirosis can also occur in temperate climates and in travelers returning from tropical areas.[1]

Although the actual numbers are uncertain, it is estimated that are 0.1-1 per 100,000 human cases per year in subtropical climates and as high as 10 or more per 100,000 human cases per year in tropical climates.[12]

In the United States, approximately 100-200 cases of leptospirosis are diagnosed yearly. Of these cases, almost 50% of the cases are diagnosed in Hawaii; and, the mean annual incidence is 1.29 cases per 100,000 in Hawaii.[19] Because of the low incidence of leptospirosis, leptospirosis is not a reportable disease in the United States.[8]

In contrast, the true incidence of human leptospirosis in India is not known; however, there have been several studies examining rates. One study found leptospiral serovprevalance rates of 8.8% and 21.7% in Chandigarh and Varanasi, respectively.[20] A different study carried out in Kolkata, West Bengal found 23.8% of the study participants (hospitalized jaundice patients) were seropositive for leptospirosis (with pomona as the most prevalent serovar in the participants).[21]

During outbreaks and with high-risk (occupational and recreational) groups, incident rates may be over 100 cases per 100,000.[1] However, in many instances, these figures may be underestimated due to underreporting of leptospirosis cases during times of severe flooding from natural disasters for example, hurricanes).[12] For example, natural disasters including monsoons and floods increase the risk of exposure to leptospires through contact with contaminated water and mud.[19]

The risk of leptospirosis can become further widespread during rainy seasons in spring and summer. During periods of flooding, leptospirosis may cause severe outbreaks among individuals exposed to the leptospiral contaminated waters.[12]

Also, during harvest times, there can be outbreaks of leptospirosis due to increased contact with infected rat populations.[10] It is estimated that 20% of all rats carry leptospires that can infect humans; although, this carrier rate can vary depending on the geographic area.

It has also been estimated that a comparable percentage of “rat-friendly” areas (urban pond, slow-moving rivers and canals, and lakes near farms) are contaminated with leptospires due to contamination from the rats.[9] Because leptospires cannot replicate outside their hosts, slow-moving water, small pools of water, and underground water are at higher risk of leptospire contamination than larger, faster bodies of moving water which can dilute the number of leptospires and decrease the risk of an individual coming into contact with leptospires.[14] Outbreaks of leptospirosis can also be associated with altered animal reservoir populations, changes in human behavior (moving into new areas), sewage contamination, and natural disasters.[1]

Depending on the area of the world, case fatality rates for leptospirosis range from <5% up to 30%.[1] These figures have been improved with the use of hemodialysis during renal failure and aggressive supportive care.[1] However, the highest mortality rates remain among the elderly and those with Weil's syndrome.[11]

## PREVIOUS OUTBREAKS OF LEPTOSPIROSIS

Since the original identification of leptospirosis by Dr. Weil,[22] outbreaks of leptospirosis have occurred sporadically throughout world. Several outbreaks of leptospirosis have occurred on Andaman and Nicobar islands and in southern and western parts of India since the first case of leptospirosis in India was reported by Taylor and Goyal in 1929.[22] The original outbreak in 1929 occurred among bund construction workers in a village on South Andaman.[23] Since the 1980s, there have been increasing numbers of leptospiral cases reported in the states of Orissa, Maharashtra, Karnataka, Tamil Nadu, and Kerala.[24]

Leptospirosis outbreaks frequently occur during times of flooding and natural disasters. Notable outbreaks include (1) 186 U.S. Army troops in the Panama Canal zone in 1961 who had been in a jungle exercise 10-13 days earlier, (2) Italy in 1984 due to a contaminated water fountain, (3) Thailand from 1995 through 2000, (4) Nicaragua in 1995, (5) Peru and Ecuador during heavy flooding in spring of 1998, and (6) 110 cases in triathletes (from swimming in contaminated Lake Springfield) in June and July of 1998 in Illinois and Wisconsin in the United States.[8,11,25,26]

During the Eco-Challenge-Sabah 2000 multisport expedition race in Borneo, Malaysia, several participants from multiple countries were infected with leptospirosis. The diagnosis was based on epidemiological evidence and initial screening with the Dip-S-Ticks assay.[27]

Following the Mumbai flood in July 2000, a study of 53 children <12 years of age admitted to a pediatrics department were tested for leptospirosis. Serological results indicated that almost one-third of the children had acute leptospirosis.[28]

In 2003, there were at least two serious outbreaks of leptospirosis in India. In Kerala, 616 individuals had developed leptospirosis and as many as 115 persons had died from it.[29] In September of 2003, 24 people died from leptospirosis in South Gujarat within 1 week. These deaths were from the 177 individuals infected with leptospirosis from 131 villages; and, in fact, there had been unconfirmed reports that over 500 people were ill.[30]

In February of 2004, 44 individuals were affected with leptospirosis in Tamil Nadu. There were no reported deaths.[31] However in September of 2004, there were 550 confirmed cases of leptospirosis and at least 75 deaths in South Gujarat.[32] Elsewhere in the world in 2004, a professor from the University of Hawaii and his graduate student were infected with leptospirosis while cleaning out his laboratory that had been flooded by an overflowing stream.[19]

In 2005, after heavy flooding in Maharashtra state, there were at least 100 deaths from leptospirosis within a two-day period.[33,34] Elsewhere in the world in 2005, there were at least 27 cases and eight deaths due to leptospirosis in the Ukraine. Concurrent with these human cases, a significant percentage of the local mice populations, up to 25%, were testing positive for leptospirosis.[35]

In the Dominican Republic at the end of 2005, there were four deaths due to leptospirosis. These cases were attributed to problems with sanitation and problems connected to municipal garbage collection.[36] In addition, there were at least six deaths reported due to leptospirosis in Guyana at the end of 2005. They did not find any particular pattern to the cases; and, the first death had actually occurred prior to the start of heavy rains and flooding in the country.[37]

In April of 2006, there was a rare outbreak of leptospirosis in Tajikistan. There had not been an outbreak of leptospirosis in the country in more than a decade. At least 15 cases of leptospirosis occurred; and, these were attributed to recent work on the public water system.[38] Then, in October of 2006, two clinically diagnosed cases of leptospirosis were detected in the United Kingdom. The two individuals had been on a fishing holiday in the Picardie region of northern France.[39]

In 2007, there were nearly 400 cases and at least five deaths attributable to leptospirosis in the Santa Fe province of Argentina. This outbreak followed the severe storm that pelted the region in April 2007.[40] In addition, there were at least five cases of leptospirosis in Hong Kong, China and at least six deaths due to leptospirosis over a three-month period in Sri Lanka in 2007. There were also at least three cases of leptospirosis in the Ukraine. These cases were attributed to an increase in rats.[41–43]

In January of 2008, there were concerns about a leptospirosis outbreak in Jamaica after three high school students were hospitalized with symptoms of leptospirosis.[44] There have also been recent outbreaks of leptospirosis in New Caledonia. In fact, New Caledonia has one of the highest rates of leptospirosis in the Pacific region; and, leptospirosis is even more common that tuberculosis, salmonellosis, hepatitis A, hepatitis B, and gonorrhea in New Caledonia.[45]

Also, in 2008, a new species of leptospirosis, *Leptospira licerasiae*, was discovered in the Peruvian Amazon. It may be responsible for up to 40% of the cases in the area and is an important cause of leptospirosis in the Peruvian Amazon region of Iquitos. In clinical testing of 881 patients with fever in the region, 41% had antibodies that only reacted with this new bacterium.[46] This leptospiral species is unique at the genomic level and has novel biological characteristics. Based on phylogenetic analysis, this species is considered part of the intermediate clade (between the pathogenic and nonpathogenic *Leptospira* clades); however, it does share some characteristics with the pathogenic serovars. For example, this new species is sensitive to 8-azaguanine.[47]

## HIGH-RISK EXPOSURES FOR LEPTOSPIROSIS

Occupational and recreational exposures account for 30-50% of leptospirosis cases.[11] Typically, individuals who work outdoors or with animals are at risk of the disease. Previously, most cases were due to occupational exposure. However, more cases are occurring due to recreational exposure; although, occupational exposure is still a significant concern.[1,11] Prime examples of high-risk occupational groups include pet shop owners, veterinarians, veterinary techs, agricultural workers, abattoir workers, plumbers, meat handlers, meat inspectors, hunters, laboratory staff, butchers, herders, meat carriers, coal miners, construction workers, fish industry workers, military personnel, civil emergency personnel, sewer workers, and garbage collectors.[1,8,11,12,14,26,48]

Leptospirosis has been known by a variety of names that reflect the method (usually occupational) by which the disease was contracted. Alternative names for leptospirosis include rice field fever (in Indonesia), seven-day fever (in Japan), cane cutter's disease (in Australia), swineherd's disease, dairy farm fever, swamp fever, Fort Bragg fever (in the United States), and mud fever.[1,11,26]

As for recreational exposures, high-risk recreational exposures for leptospirosis include adventure traveling to the tropics, cave explorers, camping, hiking and riding trail-bikes through contaminated puddles, swimming, canoeing and kayaking, fishing, water skiing and windsurfing, and other outdoor sports played in infected water.[1,11]

In addition, urban dwellers are also at increased risk because of potential exposure to infected rats. Also, children can be at risk by playing in infected water or having contact with infected animals.[11,48]

## TRANSMISSION OF LEPTOSPIRES

Damp alkaline soil, vegetation, and mud with a temperature >22°C promote leptospire survival and increase the likelihood of leptospiral transmission.[11] After water or soil has become contaminated with leptospires, the bacteria can survive (but not replicate) for weeks to months without an animal or human host in the mud or water.[1,14]

Animals commonly become infected with leptospires through contact with this contaminated water and soil. In addition, animals may acquire leptospires via congenital or neonatal infection and through sexual contact (i.e., rats, cattle, pigs, and dogs).[1]

Newly borne animals are typically protected from leptospirosis due to maternal antibodies. However, this immunity wanes after the first 6 months; and these animals are at high risk of infection if not vaccinated.[14,16] Also, if a fetal animal is infected in the womb and survives, it will typically become a chronic carrier of leptospires.[14] For this reason, pregnant livestock must be vaccinated in most countries after the first trimester to allow the fetus to develop antibodies to leptospires.[14]

Animals that are chronic carriers may continue to shed leptospires, either continuously or intermittently, for a few months to several years. These chronic infections may be localized in the kidneys or genital tract of the animals without any detectable symptoms. Until proven otherwise, animals should be considered potential sources of leptospires.[1,13]

Infected rodents are typically the source of human infections. These human infections are most commonly due to contact with water or environmental surfaces contaminated with infected urine.[13,49] Other rodent body fluids (excluding saliva) can also transmit leptospires to humans, animals, and the environment.[13]

The leptospires can readily enter through damaged skin and mucous membranes of the eyes, nose, and mouth.[12] Individuals can also become infected with leptospires by drinking contaminated water or eating contaminated food.[1,13] Rarely, leptospires can be inhaled from infected urine droplets.[1] Very infrequently, there may be human-to-human transmission through sexual contact, laboratory accidents, transplacentally, and through breast milk.[1,11,12]

Blood and urine from infected humans should be considered infectious for the time prior to symptom until the seventh to tenth day of illness.[1] Usually after 1 week, antibodies to the leptospires appear and clear the leptospires from the blood; however, protected body sites such as the eyes may continue to harbor leptospires for prolonged periods.[1]

## PATHOPHYSIOLOGY OF LEPTOSPIROSIS

Leptospirosis has symptoms that may mimic better-known diseases including influenza, dengue fever, meningitis, hepatitis, and other viral hemorrhagic diseases.[1,18] With the greater publicity of these other diseases, leptospires are frequently overlooked as a source of disease.[1]

Leptospirosis should be considered in the differential diagnosis of individuals who have an abrupt onset of fever, muscle aches, jaundice, headache, conjunctival suffusion, and chills. Conjunctival suffusion and muscle aches in the calf and lumbar areas are also highly characteristic of leptospirosis; however, these signs and symptoms are not confirmatory for leptospirosis.[1]

During the course of infection, leptospires invade all the internal organs and tissues, and damage the endothelial linings of the small blood vessels. Glycoprotein toxin produced by the leptospires causes capillary leakage which can result in severe hemorrhaging.[50] This damage is the source of the majority of the signs and symptoms of leptospirosis.

As the damage progresses, lesions develop throughout the organs. This damage results in (1) injury in the proximal convoluted tubules (leading to interstitial nephritis) in the kidneys, (2) hepatic capillary damage with hepatic cell damage leading to jaundice, blood-clotting problems, and possible liver failure, and (3) inflamed meninges resulting in the symptoms of aseptic meningitis in the immune phase.[1]

## IMMUNE RESPONSE TO LEPTOSPIROSIS

The antigenic structure of leptospires is complex. The outer membrane is composed of lipopolysaccharide and is highly immunogenic. The outer membrane is serovar-specific; and it is the primary target of both humoral and cell-mediated immunity.

In contrast to the outer membrane antigen, the somatic antigen is genus-specific; and, the flagellar antigen is both genus and serovar-specific. Further, some serovars, (i.e., *L. icterohemorrhagie*), have an antigen associated with virulence termed, Vi.[26]

After infection with leptospires, macrophages begin to ingest the leptospires which is then aided by the production of protective antibodies as the infection progresses. These anti-leptospire antibodies may be directed against antigens to all leptospires or to antigens specific to a particular serovar or serogroup.

Usually antibody production begins within 5-7 days after infection; however, antibody production may take 10 days or longer, especially in immunocompromised individuals.[1] The IgM antibodies usually appear before the IgG antibodies. These IgM titers may persist at low levels for months and possibly for years after the infection. In contrast, the IgG titers may remain only transiently or persist for years.[1]

Concurrently, IgA begins to appear on approximately the fifth day of infection and may persist for as long as 9 months. Finally, cell-mediated immunity assists in preventing leptospiral colonizaton in the renal tubles.[26]

Some individuals may initially produce antibodies that cross-react with multiple serovars. These cross-reactive antibodies usually appear in the early stages of the disease and disappear as the disease progresses.

Antibodies specific to a particular genus may persist for months. In contrast, the serovar-specific antibodies may persist for years.[1] These serovar-specific antibodies are considered protective against reinfection with that serovar; however, they will not necessarily prevent infection with other serovars.[1]

## SIGNS AND SYMPTOMS OF HUMAN LEPTOSPIROSIS [TABLE 2–4]

**Table 2:** Potential septic stage symptomatology

High fever
Chills with rigors
Persistent headache
Extreme exhaustion
Photophobia
Conjunctival bleeding
Conjunctival suffusion without purulent discharge
Pharyngeal injection
Hemoptysis
Cough and possible respiratory problems
Chest pain
Cardiac arrhythmias
Decreased blood pressure with possible circulatory collapse
Anemia
Swollen lymph nodes
Lack of appetite
Nausea of vomiting
Abdominal pain
Mild jaundice
Enlarged liver and spleen
Skin rash
Severe muscle pains (primarily affecting the calves, back, and abdomen)
Joint pain

**Table 3:** Additional immune stage symptomatology

Altered mental status
Uveitis
Iritis
Iridocyclitis
Chorioretintis
Cough with respiratory distress
Congestive heart failure
Pericarditis
Hemorrhages
Acalculous cholecystitis primarily seen in pediatric cases

**Table 4:** Possible clinical abnormalities associated with leptospirosis[15,24,34]

Altered cerebrospinal fluid (CSF) cell count (usually less than 500/mm) with Polumorpho-nuclear cells seen early in disease and mononuclear cells are seen during late infection.
Altered CSF protein levels: Less than 40 mg/dl (normal) to 300 mg/dl
Normal CSF glucose
Non-specific ECG changes
Highly elevated serum creatinine phosphokinase (CK) and MB variant
Elevated erythrocyte sedimentation rate (60 mm)
Normal or reduced serum potassium
Elevated serum bilirubin levels
Elevated serum glutamic oxaloacetic transaminase, serum glutamic pyruvic transaminase and alkaline phosphatase
Possible elevated serum amylase
Low to normal erythrocyte count and hemoglobin
Thrombocytopenia
Possible elevated prothrombin times
Leukocytosis with increased neutrophils
Elevated creatinine phosphokinase and blood urea nitrogen: Less than 100 mg/dL (typically) to above 300mg/dl (rarely)
Patchy alveolar pattern in the peripheral portions of the lower lung lobes due to alveolar hemorrhage
Azotemia and oliguria/anuria typically during the second week of illness
Proteinuria
Hyaline or granular casts
Hematuria
Pyuria

There are two clinically recognizable syndromes produced by leptospiral serovars. The first syndrome, anicteric leptospirosis, is composed of two stages known as the septic (septicemic) stage and the immune stage. The second more severe syndrome is known as icteric leptospirosis or Weil's syndrome.[26] Increasing age and multiple chronic health conditions can increase the severity of these syndromes and the potential for death.[1]

Approximately 85-90% of leptospirosis cases are anicteric leptospirosis. The typical incubation period is 7-12 days (with a range of 2-20 days). This form of leptospirosis is usually self-limiting.[26]

During the septic stage, which normally lasts 3-7 days, there may be an abrupt onset of a variety of signs and symptoms.[1,11,26]

These symptoms are most noticeable during the 4th through 17th days following infection then the fever normally decreases as bacterial lysis occurs. During the septic stage, leptospires can found throughout the body and can be isolated from the blood and cerebrospinal fluid (CSF).[11,26]

Prior to the onset of the immune stage, there is a one- to three-day period with minimal symptoms. Symptoms then reemerge in the immune stage. This immune stage typically lasts 4-30 days. In the immune stage, IgM antibodies begin to appear in the blood. Many of the earlier symptoms return (i.e., fever, headache, and vomiting) but are decreased.

Typically, the leptospires are cleared from the blood and CSF within the first few days of the immune stage; however, leptospiruria (i.e., leptospires in the urine) will begin as leptospires settle in the brush border of the proximal convoluted tubles and begin to replicate and be released in the urine. This leptospiruria will typically persist for 1-3 weeks.[1,26]

In addition, aseptic meningitis, which is the hallmark of the immune stage, will usually occur and last a few days up to 1-2 weeks.[11] Other signs and symptoms that may occur during the immune stage.[11,26,50]

In contrast to anicteric leptospirosis, icteric leptospirosis or Weil's syndrome is a much more severe syndrome; although, only 5-10% of leptospirosis cases are Weil's syndrome.[19] According to the literature, Weil's syndrome has a fatality rate of 5-10%; and, the rate increases to 20-40% with hepatorenal involvement and jaundice.[11]

Weil's syndrome is characterized by liver, kidney, and vascular dysfunction in addition to the other symptoms of anicteric leptospirosis. Individuals with Weil's syndrome will usually develop jaundice without hepatocyte destruction and azotemia by the third to seventh day of illness.[26]

The liver may be enlarged and there may be right upper quadrant tenderness. With increasing severity of jaundice, the individual is at greater risk of developing renal failure, hemorrhage, and cardiovascular collapse.[11]

Uremia, oliguria, and anuria may occur with the onset of kidney failure unless supportive treatment (i.e., dialysis) is provided. Fatalities due to icteric leptospirosis are typically due to renal failure, cardiopulmonary failure, and fatal hemorrhages.[1]

Most individuals recover from leptospirosis within 6-12 weeks after the onset of illness without further sequelae. However, full recovery may take years. Even with full recovery, there is no cross-protective immunity to other leptospiral serovars.[51]

In approximately 10% of cases, there can be long-term effects after leptospirosis. If the acute phase was not properly diagnosed, the long-term effects may not become apparent until later medical treatment which may still be misdiagnosed.

Chronic sequelae of leptospirosis may include (1) recurrent migraines, (2) eye pain, (3) uveitis and iridocyclitis, (4) chronic tiredness, (5) depression and mood swings, (6) obsessive-compulsive disorder, (7) paresis and paralysis, and (8) parainfectious encephalomyelitis.[1,8,10,48,52] These effects may be due to leptospires persisting in protected regions of the body (i.e., the eyes and brain). A study in India was able to isolate leptospires from the eyes of patients with these sequelae.[52] Although there is no confirmed treatment, repeated courses of antibiotics have been shown to eliminate the remaining leptospires in the protected areas of the body. It is recommended that leptospirosis patients be monitored for sequelae during the 5 years after recovery.[52]

Any serovar may produce severe disease; and, the infectious dose may affect the severity of illness.[1] In fact, simultaneous infections from multiple serovars are possible though not as widely reported as expected.[50]

Some signs and symptoms are more common with particular serovars. For example, jaundice occurs in 83% of individuals with serovar icterohemorrhagiae infection and in 30% of individuals with serovar pomona infections.[11] Serovar autumnalis infections produce a unique pretibial erythematous rash; and, serovar grippotyphosa produces significant gastrointestinal symptoms. Further, infections with serovars pomona and canicola are more likely to produce aseptic meningitis.[11] Weil's disease is usually associated with the rat-transmitted serovars icterohemorrhagiae, bataviae, lai, and copenhageni.[1,50]

Finally, during pregnancy, leptospirosis may cause fetal death, abortion, stillbirth, and/or congenital leptospirosis.[1] Further, there is an increased rate of spontaneous abortion if the infection occurs in the first trimester of pregnancy.[11]

## DIFFERENTIAL DIAGNOSES OF LEPTOSPIROSIS [TABLE 5]

**Table 5:** Differential diagnoses of leptospirosis

Influenza	Dengue
Hantavirus	Yellow fever
Viral hemorrhagic fevers	Rickettsiosis
Borreliosis	Brucellosis
Malaria	Pyelonephritis
Septic meningitis	Lyme disease
Chemical poisoning	Food poisoning
Typhoid fever	Enteric fevers
Viral hepatitis	Fever of unknown origin
Primary HIV seroconversion	Legionnaire's disease
Toxoplasmosis	Infectious mononucleosis
Pharyngitis	-

Because leptospirosis has nonspecific symptoms that can mimic more familiar diseases, a careful history needs to be done.[1,50] Special attention to occupational and/or recreational exposures to potentially contaminated animals and environments should be considered.[1]

## SAMPLE COLLECTION OF LEPTOSPIRES [TABLE 6]

**Table 6:** Useful methods for sampling of leptospires[15,35]

Blood with added heparin within the first 10 days of infection
Paired sera collected several days apart: Based on the date on onset of illness and the probable time of seroconversion
Fresh urine less than two hours old is needed as Leptospires die quickly in urine
Organ and tissue samples for serology
Cerebrospinal fluid Within the first 10 days of infection
Dialysate for culture

Leptospires can survive in anticoagulated blood for as long as 11 days which allows blood samples to be mailed to reference laboratories for culture.[11] In addition, leptospires will survive better in tissues held at 20°C and above. Otherwise, the leptospires will begin to die as the alkalinity declines with cellular breakdown. However, tissues should not be frozen because freezing will reduce leptospire viability.[1]

As for urine, leptospires may be isolated for weeks and possibly years after the initial infection; however, the urine cultures may take as long as 8 weeks to grow leptospires.[11]

## DIAGNOSIS OF LEPTOSPIROSIS

Laboratory testing is necessary to confirm the diagnosis of leptospirosis and to determine the serovar of the leptospires. By determining the serovar, it can provide an indication of the source of the infection and possible effective control measures that should be implemented.[1,12]

There are a variety of tests that can be used to detect leptospires. These testing methods may either directly visualize leptospires or indirectly detect leptospires through serological methods. However, isolation of pathogenic leptospires is the only definitive proof of infection.[1]

### Direct detection of leptospires

Leptospires can be directly detected via cultures, inoculation of experimental animals, polymerase chain reaction (PCR), (immuno) staining, DNA homology studies, restriction enzyme analysis (REA), and dark-field microscopy.[1]

Dark-field microscopy can be used to visualize leptospires; however, this method cannot assist in differentiating pathogenic leptospires from nonpathogenic saprophytic leptospires or in differentiating the pathogenic leptospire serovars.[1] Further, artifact on the slide can be mistaken for leptospires and correct identification of leptospires is dependent on the technical expertise of the individual using the microscope. Therefore, dark-field microscopy should not be a first choice for diagnosing leptospirosis.[11]

DNA homology studies are used to classify leptospire strains into species. Approximately 300 strains have been classified using DNA homology. DNA homology studies use quantitation of DNA-DNA hybridization to measure DNA relatedness among leptospiral strains.[1]

Restriction enzyme analysis (REA) and pulsed-field gel electrophoresis (PFGE) can be used more readily than DNA homology studies to differentiate leptospires to the subspecies level. Ribotyping and PCR can also be used for typing leptospires.[1]

### Indirect detection of leptospires

Because the existing direct detection methods for leptospires are unreliable and slow, serology can more effective for diagnosis. There are a variety of rapid screening tests for leptospirosis (that primarily use serology for detection); however, the presence of antibodies is not proof of a current infection therefore the results (both positive and negative) should be confirmed by further testing (especially with the microscopic agglutination test, MAT).[1,11] Examples of rapid screening tests include the IgM enzyme-linked immunosorbent assay (ELISA), macroscopic slide agglutination test, the Patoc-slide agglutination test, the microcapsule agglutination test, latex agglutination tests, dipstick (Dip-S-Ticks) tests, and the indirect hemagglutination test.[1,11]

ELISA testing can be used with a genus-specific antigen to detect IgM and sometimes IgG antibodies. ELISA tests can be useful for screening new-onset infections especially because these genus-specific tests are more likely to be positive earlier in the illness than the MAT.[1,11] However, the genus-specific antigen is shared by both pathogenic and saprophytic leptospires; and, therefore, it cannot indicate the infecting serovar.[1]

Seroconversion of a negative first sample to a positive second sample or a fourfold or higher rise in titer can be considered diagnostic of a recent or current infection.[1] A high IgM titer with ELISA can be indicative of a current or recent infection; however, IgM levels can remain detectable for several months to years so elevated IgM levels cannot positively confirm a diagnosis of leptospirosis.[1]

The ELISA can be standardized unlike the MAT; however, the ELISA is less specific than the MAT and can cross-react with other diseases.[1] Further, there can be difficulties in interpretation of test results with low titers.

Low titers can be problematic in endemic areas because of probable prior exposures to leptospires. A low titer in an endemic area may only be indicating a previous infection or it may be indicating that (1) the clinical sample was taken at an early or late stage of infection, (2) the infection is extremely severe, (3) the individual is immunosuppressed, or (4) high doses of antibiotics have been administered. In contrast, low titers are likely to be indicative of infection in nonendemic areas.[1]

The macroscopic slide agglutination test uses killed antigen to screen individuals for possible leptospirosis; however, this test is not specific and requires confirmatory testing.[11]

The Dip-S-Ticks assay has been used successfully by the CDC for initial screening for leptospirosis. Evaluations at the CDC indicate that the Dip-S-Ticks assay appears to have greater sensitivity than other assays during the early stages of infection. With the Dip-S-Ticks assay, the sensitivity is approximately 27% on the third day after the onset of symptoms. This sensitivity increases to 84% by the seventh to ninth day after symptom onset; and, it is nearly 100% by the 10th to 12th days.[27]

### Microscopic agglutination test

The gold standard for serodiagnosis of leptospirosis in both animals and humans is the MAT.[14] The MAT is currently the most effective test in determining the serovar and serogroup of leptospires. High specificity is the major advantage of the MAT.[1]

The MAT determines agglutinating antibodies in the serum or urine of a patient by mixing in samples of live or killed leptospires. Numerous serovars of leptospires must be maintained under laboratory conditions to perform the MAT. This limits the availability of the MAT and makes the testing labor intensive.[1,8,48]

In addition, a saprophytic strain (*L. biflexa* strain Patoc I) is included in the testing because this strain cross-reacts with human antibodies produced against several pathogenic serovars and assists in determining if the reaction is nonspecific.[1]

The MAT consists of two consecutive steps. The first step screens for the responsible serogroup; and, the second step quantitatively determines the serum titer for every test antigen. The MAT is usually positive 10-12 days after symptom onset; although, antibodies are usually present by 5-7 days after symptom onset.[1]

If antibodies (either IgM or IgG) to the particular serovar are present, agglutination will occur and be observable using dark-field microscopy.[1] During the initial stages of the infection, cross-reactive antibodies to multiple serovars may be present in the serum which may affect the results of the MAT. These cross-reactive antibodies decrease through the progression of the illness as the titer of homologous antibodies increases.[1]

The MAT cannot differentiate between antibodies from current, recent, or past infections. Therefore, it is most effective to test two consecutive serum samples for a fourfold or higher increase in the titer.[1]

Further, there are several diseases and conditions that can produce antibodies that will cross-react in the MAT. Examples of these diseases include legionellosis, hepatitis, and autoimmune diseases.[1]

In addition, the MAT may not provide a positive reaction to a previously unknown leptospire. Further, it cannot be standardized because live leptospires are used. Instead, paired serum samples should be analyzed together.[1]

Finally, monoclonal antibodies can be used to type leptospires. The monoclonal antibodies can be used in the MAT to react with single antigenic characteristics (epitopes) on the outer surfaces of the leptospires. These epitopes may be specific for a particular serovar or shared by several serovars. This allows the identification of leptospires to the serovar level and occasionally to the subserovar level. Monoclonal antibodies can also detect differences within strains of the same serovar. In this manner, new serovars may be indicated by differences in agglutination patterns obtained with a panel of monoclonal antibodies.[1]

### Cultivation of leptospires

Leptospires can be cultivated in a variety of media; however, strict sterility must be maintained during culture media preparation and work with the clinical sample because nonpathogenic saprophytic leptospires can readily contaminate the sample.[1]

The essential nutritional requirements for leptospires are Vitamin B1 and B12 and long-chain fatty acids (complexed with albumin). Further, pyruvate assists in initiating growth in leptospires.[1]

Leptospires do not require external sources of pyrimidine bases for incorporation into their genomes. This makes leptospires resistant to the antibacterial action of 5-fluorouracil. By adding 5-fluorouracil to media, the media can be made selective for leptospires.[1]

Leptospires take approximately 6-8 h or more to double in culture media. Optimal growth occurs at 28-30°C; although, some serovars are more fastidious. The culture needs to be checked weekly for up to 4 months using dark-field microscopy to detect growth.[1]

## LABORATORY SAFETY FOR LEPTOSPIROSIS

Standard laboratory safety procedures are necessary to work with leptospires. The main concern with leptospires is accidents which may damage the skin (cuts, abrasions, punctures, and bites) and expose the eyes. Leptospires can be destroyed by desiccation, acid, phenolic compounds, disinfectants and antiseptics, and heat. Prophylactic antibiotics should be given if there is a possible exposure. Because of potential exposures to other infectious agents, laboratory workers should be vaccinated against hepatitis B, leptospirosis, and rabies as necessary.[1]

## TREATMENT OF LEPTOSPIROSIS [TABLE 7]

**Table 7:** Dosing regimens for leptospirosis[11,27,50,53]

**Penicillin G**
Adult dose: 20-24 million U/d IM divided q4-6h
Pediatric dose
- Children under lbs: 600,000 U IM
- Children from 30 to 50 lbs: 900,000 to 1.2 million U IM
- Children over 50 lbs: Administer as in adults
**Crystalline penicillin**
Dosage: 6-8 megaunits/day divided IV and IM
**Procaine penicillin and crystalline penicillin**
Beginning dosage: 2-2.5 megaunits/day each IM
Reduce to 1-1.2 megaunits/day each IM when fever reduces, totaling a 5-7 days course
Then 1.5 megaunits/day of procaine penicillin until 2 days after cessation of albuminuria
**Erythromycin**
Adult dose: 500 mg IV QID
Pediatric dose: 30-50 mg/kg/d in 3-4 divided doses
**Amoxicillin**
Adult dose: 500 mg PO QID
Alternatively: 1 g IV if severe leptospirosis
Pediatric dose: 20-40 mg/kg/day
**Ampicillin**
Adult dose: 500-750 mg PO QID
Alternatively: 1 g if severe leptospitosis
Pediatric dose: 50 mg/kg/day
**Doxycycline**
Adult dose: 100 mg PO BID
Pediatric dose:
- Children under 8 years: Not recommended
- Children over 8 years: 2 mg/lb PO divided BID on first day, followed by 1 mg/lb/d qd or divided BID on subsequent days
Pre-exposure prophylaxis: 200 mg once per week (95% efficacy against leptospirosis[11]
Post-exposure prophylaxis: 100 mg per day for 7 days
**Tetracycline**
Initial dose of 500 mg
250 mg every 8 h, PO, IV or IM for 24 h
Then, 250-500 mg QID for 6 days
**Caftriaxone**
1.0 g q 24 h for 7 days

For mild leptospiral infections, treatment is primarily based on the symptoms. Rest and rehydration are encouraged because increased activity can potentially prolong or worsen the illness. In addition, non-aspirin-based analgesics and antibiotics may also be used.[50] Milder cases of leptospirosis can be treated with amoxicillin, ampicillin, doxycycline, penicillin, streptomycin, tetracycline, or erythromycin. Ceftriaxone and cefotaxime, and quinolone antibiotics may also be used for treatment of mild leptospirosis cases.[1,11,51]

Treatment with antibiotics should begin as soon as leptospirosis is suspected and if possible before the fifth day after the start of symptoms. However, serological tests will not be positive until about 1 week after the onset of symptoms and cultures may not be positive for several weeks.[1]

For individuals with severe infections and Weil's syndrome, hospitalization in an intensive care unit is typically required. Intravenous penicillin should used for severe leptospirosis.[1] Dialysis will likely be necessary for the renal failure. Transfusions may be needed for hemorrhaging; and, steroids may be necessary for treatment of thrombocytopenia.[50]

As an additional note, Jarisch-Herxheimer reactions may occur within 4-5 h of administration with penicillins. With the reaction, the patient's temperature rises to 37.8-38.4°C and is accompanied by severe rigors and hypotension. The reaction is temporary and does not require the stopping of therapy with penicillins.[50]

## PREVENTION AND CONTROL OF LEPTOSPIROSIS [TABLE 8]

**Table 8:** Preventive measures for leptospirosis

Animal surveys of a locale
Animal surveys of an area help determine the reservoir of the leptospires and examine the size of the population of these host animals. In addition, culturing of kidney tissue from these host animals can help detect the leptospiral serovars in a particular area
Water and soil samples
These studies are primarily retrospective and not hightly effective in controlling leptospirosis
Rodent control
Installation of fences and screens to exclude rats and feral animals
Rodent-proofing of buildings
Removal of food and trash from recreational areas which may attract rodents and other feral animals
Trapping and poisoning of rats and feral animals
Warning signs for potentially contaminated areas
Limiting access to potentially infected water sources and mud
Hygiene and sanitation
Provision of safe drinking water
Protective clothing (gloves, boots, glasses, aprons, and masks)
Sanitary procedures (washing hands)
Washing and (occlusive) bandaging of wounds
Avoidance of potentially contaminated water and soil
Avoidance of potentially contaminated body fluids and tissues
Antibiotic prophylaxis
Vaccines
Litter control
Disinfection and cleaning
Disinfection of contaminated areas (barns, slaughterhouses)
Cleaning with antibacterial cleaning solution or a solution of 1 part household bleach in 10 parts water
Bleach, acids, alkalis, chlorines, disinfectants, UV filtration, steam-cleaning, irradiation, and drying will typically kill leptospires
Harvesting
Use machinery when possible for harvesting thus reducing human contact with potentially infected animals and environmental sources
Prevention of domestic livestock
Buying certified leptospire-free animals
Avoiding communal pastures if possible
By keeping sheep and infected animals out of a pasture for at least 2 months will usually provide sufficient time for any leptospires present in the field to die because drying and sunlight will kill the leptospires
Isolation and slaughtering of infected animals
Antibiotics for potential leptospite sheeding
Vaccines
Serovars icterohemorrhagiae and canicola for dogs
Serovars hardjo and pomona for cattle
Serovars pomona, tarassovi and bratislava for pigs
Proper disposal of animal waste to prevented infection in uninfected animals and humans
Effective laboratory safety procedures
Education
Causative organism
Signs and symptoms
Control and prevention measures
Treatment

For proper prevention of leptospirosis, the preventive measures must be based on the individual's risk factors and epidemiological factors. Preventive measures may include[1,10,12–14,19,17]: 

### Vaccination against leptospirosis

Most of the vaccines on the market to prevent clinical disease caused by leptospires are designed for use in animal species rather than in humans. The vaccines are effective only against the serovars included in the particular vaccine; and, further, these vaccines may not prevent infection or the development of a carrier state in animals.[1,11,13,15]

It has been found that bacterin-derived vaccines (i.e., vaccines created to immunize against bacterial diseases) against leptospirosis and other diseases (for example, kennel cough and Lyme disease) probably do not even provide protective immunity for a full 12 months. Without more frequent vaccinations, this may lead to outbreaks of these diseases.[15]

Examples of current leptospirosis vaccines on the market for animals include the Duramune Leptospirosis vaccine and Spirovac^©^. Fort Dodge has available the Duramune Leptospirosis vaccine which can immunize against the serovars grippotyphosa, pomona, icterohemorrhagiae, and canicola. This vaccine is a subunit vaccine composed of only the immunogenic component of the leptospires. This can greatly reduce potential side effects while concurrently providing protection from disease.[15] Spirovac^©^ can be used to vaccinate calves as young as 4-weeks old with a repeat immunization at 4 weeks after the initial injection then repeat boosters annually.[54]

As for human vaccines for leptospirosis, there is a killed vaccine available in China, Japan, and Vietnam to prevent leptospirosis in humans.[10] However, human vaccines for leptospirosis are serovar-specific and require yearly boosters. In addition, there can be painful swelling after revaccinations.[1,11]

The vax-SPIRAL vaccine was developed in Cuba for the control of leptospirosis in human populations at risk. The efficacy of the vaccine was 78.1% (95% confidence interval (CI): 59.2-88.3%), during testing on human subjects; and, the side effects were primarily general discomfort and mild pain at the injection site.[55]

## FUTURE CHALLENGES ASSOCIATED WITH LEPTOSPIROSIS

Future challenges associated with leptospirosis include: (1) effective prevention and control, (2) improved diagnosis, and (3) improved access to treatment.

Although it is impossible to control the occurrence of natural disasters, planning can be done at all levels of government to more rapidly and effectively respond to the effects of flooding to prevent and control situations that may potentiate outbreaks of leptospirosis.

It is known that leptospires can alter their biosynthetic mechanism for the production of the lipopolysaccharides in their outer membrane thus allowing them to adapt to new hosts.[56] Further attention needs to be given to the prevention of leptospires from spreading into new host species.

Next, there needs to be continuing development of rapid (and effective) diagnostic tests that can be used immediately in areas where natural disasters have occurred rather than having to wait for confirmatory results from a limited number of laboratories.[8]

Without proper diagnosis, many cases of leptospirosis are going undiagnosed and potentially leading to chronic health effects. With proper diagnosis, individuals can be treated more rapidly and recover with less long-term disability and mortality.

Finally, there need to be continuing development of more effective and wider spectrum vaccines against the diversity of leptospiral serovars. It has been theorized that recombinant vaccines can be developed against the outer membrane proteins of leptospires thus allowing concurrent protection against multiple serovars.[56]

## LEPTOSPIROSIS RESEARCH IN INDIA

It is clearly evident from the literature from leptospirosis is reemerging as a significant source of disease in India and that these cases are not limited to a particular geographic area or subpopulation (i.e., children).[57]

One study examined the characteristics of leptospirosis in Chennai. The majority of the cases were anicteric.[58] Also, in studies of acute pancreatitis, leptospirosis accounted for approximately 10% of the cases and it was found that many patients with leptospirosis developed pancreatitis prior to the development of renal failure.[59,60] Further, in a study of SIADH, leptospirosis accounted for approximately 9% of the cases.[61] It was also justified that leptospirosis should be considered as a differential diagnosis for typhoid hepatitis and rhabdomyolysis.[62,63] Other investigations noted that leptospirosis could be the cause of many febrile illnesses in urban slums during and after the monsoon seasons.[64] When comparing several clinical and epidemiological criteria useful in the diagnosis of leptospirosis with the MAT, it was found that these criteria had a high negative predictive value that would assist in excluding leptospirosis as a cause of an illness.[65] Rainfall and exposure were defined as an important correlation in diagnosing patients with leptospirosis in India.[66]

Indian investigators proved that high-dose glucocorticoid pulse therapy (GPT) were effective in treating the pulmonary symptoms due to leptospirosis and recommended starting high-dose GPT as soon as possible after the onset of dyspnea.[67]

## THE REGIONAL MEDICAL RESEARCH CENTER IN INDIA ON LEPTOSPIROSIS

The Regional Medical Research Center on Leptospirosis is situated at Port Blair in Andaman and Nicobar Islands. It is a permanent institute of the Indian Council of Medical Research. It conducts research on Leptospirosis which is one of the diseases affecting residents of the islands. It is the National Reference Center for Leptospirosis in India and also been designated as World Health Organization Collaborative Center for Diagnosis, Reference, Research, and Training in Leptospirosis.[68]

## CONCLUSION

Leptospirosis can be labeled a truly menacing disease. It fits the spectrum of agent, host, and environment which relates to clinical diseases with a community focus.

Developing algorithms for public health training can be a good start to tackling Infectious Diseases of Public Health importance in India. Leptospirosis is just one of them.
